# Pharyngeal penetration complicated by pneumomediastinum and surgical emphysema: a rare pediatric case report

**DOI:** 10.3389/fped.2025.1638899

**Published:** 2025-11-18

**Authors:** Belal Al Droubi, Rana AlKatheeri, Esameldin Salih, Maryam Ali AlQaydi, Mohammed Alsamri

**Affiliations:** 1 Department of Pediatrics, Tawam Hospital, Al Ain, Abu Dhabi, United Arab Emirates; 2 Department of Otolaryngology, Tawam Hospital, Al Ain, Abu Dhabi, United Arab Emirates; 3 Department of Radiology, Tawam Hospital, Al Ain, Abu Dhabi, United Arab Emirates; 4 Departments of Pediatrics, College of Medicine and Health Sciences, United Arab Emirates University, Al Ain, Abu Dhabi>, United Arab Emirates

**Keywords:** pediatric trauma, oropharyngeal injury, neck injury, penetrating injury, surgical emphysema

## Abstract

Oropharyngeal injuries in young children, though common, carry the potential risk of serious complications. Here, we describe a rare case of a toddler with a minor penetrating injury in the hypopharynx leading to perforation, surgical emphysema, and pneumomediastinum. A 2-year-old boy was brought to the emergency department with noisy breathing and shortness of breath that started after a traumatic injury with a pencil in the mouth. He was found to have tachycardia, tachypnea, and grunting, but no stridor or retractions. A brief oral exam showed a blackish discoloration in the posterior pharyngeal wall, and crepitations were palpated on the neck. Imaging revealed extensive subcutaneous emphysema and a defect in the posterior oropharyngeal wall. He underwent surgical repair of the pharyngeal defect and was later discharged in stable condition. This case highlights the need for vigilance in assessing oral impalement injuries in children, as such injuries can lead to significant complications. Early detection and management are crucial to prevent severe outcomes. The case underscores the importance of careful clinical and radiological evaluation in managing pediatric oral impalement injuries.

## Introduction

Oropharyngeal trauma is a frequent occurrence in young children. This age group is more vulnerable as they often walk or run with objects in their mouths. The injury occurs when an object is forced directly into the oral cavity. The majority of these injuries are unwitnessed, heal spontaneously, and are managed conservatively if they seek medical attention (1). Impalement injury to the oropharynx has been reported to cause internal carotid artery injury and neurologic sequelae (2). Additionally, mucosal injury can introduce air into the surrounding tissues, leading to subcutaneous emphysema and pneumomediastinum. While the majority of the air leaks are benign and self-limiting, they can sometimes have severe complications, such as airway compromise, infection, and mediastinitis (3). A high index of suspicion is crucial to identify and prevent serious complications that may arise from trivial trauma.

Here, we describe a 2-year-old boy with an oral injury who was initially asymptomatic on evaluation. The occurrence of neck crepitus necessitated additional radiologic imaging and surgical assessment.

## Case presentation

This previously healthy 2-year-old boy presented to the pediatric emergency department with a 2 hours history of acute-onset noisy breathing and shortness of breath. His past medical history was unremarkable, with no previous hospitalization, chronic medications, or chronic illnesses. There was no family history of chronic respiratory conditions. The mother reported mild viral upper respiratory tract infection symptoms a few days preceding the event. Immediately before the onset of acute respiratory distress, the toddler was lying prone on the floor, scribbling with a pencil, when his sibling jumped onto him. This action forced the vertically held pencil into his mouth. The mother noticed a small amount of fresh blood in the oral cavity, but no active bleeding. The pencil was removed intact with no retained fragments. The toddler developed a transient hoarse voice immediately after the injury. Importantly, there were no episodes of stridor, cyanosis, or loss of consciousness.

On physical examination, the toddler appeared irritable but was consolable. His vital signs revealed tachycardia (heart rate 150 beats/min), mild tachypnea (respiratory rate 38 breaths/min), normal oxygen saturation (95% in room air), and afebrile temperature 37 °C. He had mild hoarseness of voice, but no neck pain, drooling, or torticollis. Respiratory examination demonstrated a noticeable grunting without stridor or subcostal retractions. Auscultation revealed bilateral wheezing, more prominent on the left side. The child was uncooperative for a complete oral examination, but a brief exam showed blackish discoloration on the posterior pharyngeal wall. Subcutaneous crepitus was palpable bilaterally in the neck. Neurological examination revealed no focal deficits.

**Figure 1:**
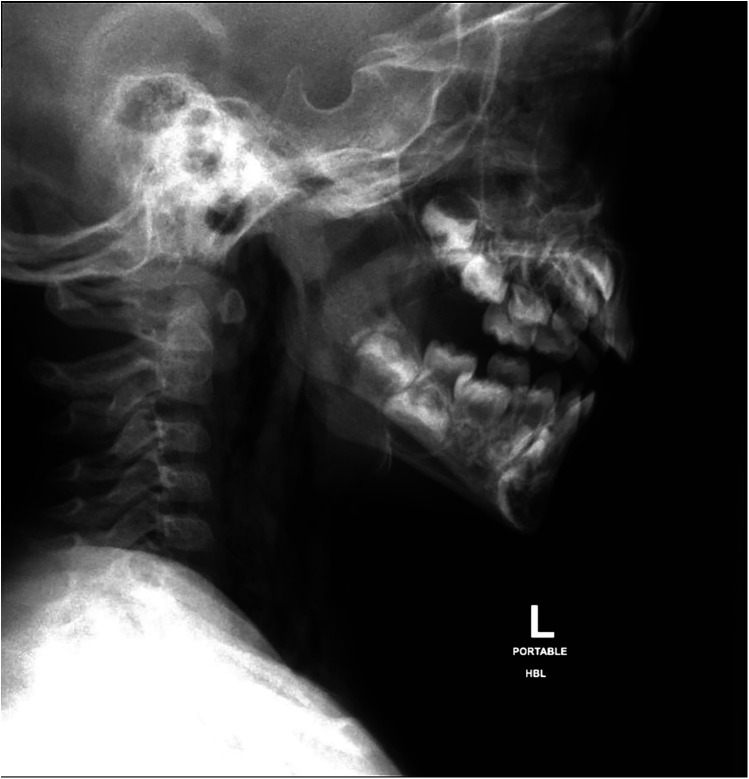
Lateral neck x-ray, showing surgical subcutaneous emphysema of the neck and dissecting down to the mediastinum.

**Figure 2:**
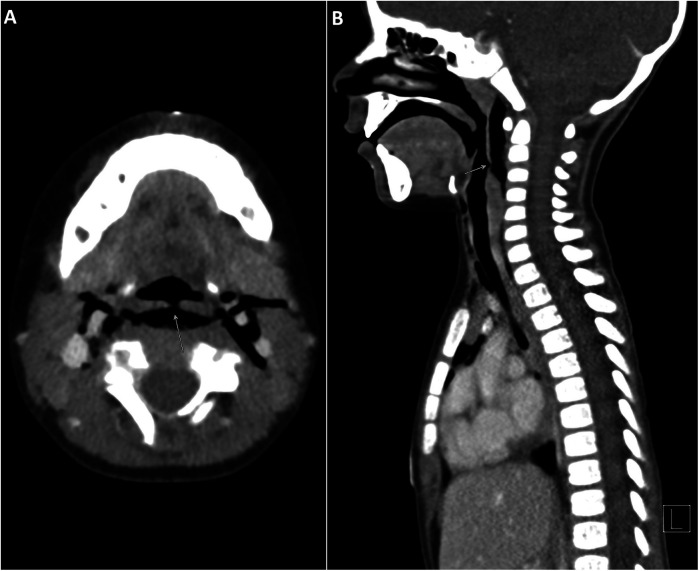
**(A)** Axial CT neck with IV contrast. A focal defect in the posterior hypopharyngeal wall (arrow). Soft tissue emphysema involves the retropharyngeal and prevertebral space and extends laterally surrounding the internal carotid arteries bilaterally. **(B)** Sagittal CT neck with IV contrast. Posterior hypopharyngeal wall defect (arrow) with extensive soft tissue emphysema tracking through the retropharyngeal and prevertebral spaces. CT, computed tomography.

Initial diagnostic imaging was performed to evaluate the extent of the injury. His chest and lateral neck x-rays showed extensive surgical emphysema in the neck extending into the mediastinum (Figure 1). A contrast-enhanced computed tomography (CT) of the neck and chest identified a posterior hypopharyngeal wall defect, with extensive soft tissue emphysema involving the retropharyngeal prevertebral space, surrounding the internal carotid sheaths bilaterally. Notably, there was no extravasation of the IV contrast or major vascular injury (Figures 2A,B). Based on these findings, a sizable pharyngoesophageal injury was highly suspected, raising major concern for the managing team.

The child was admitted to the pediatric intensive care unit and started on broad-spectrum IV antibiotics to reduce the risk of mediastinitis and local pharyngeal wound infection. Given the clinical and radiologic findings of extensive soft tissue emphysema, a significant injury to the pharynx and esophagus was strongly suspected.

**Figure 3:**
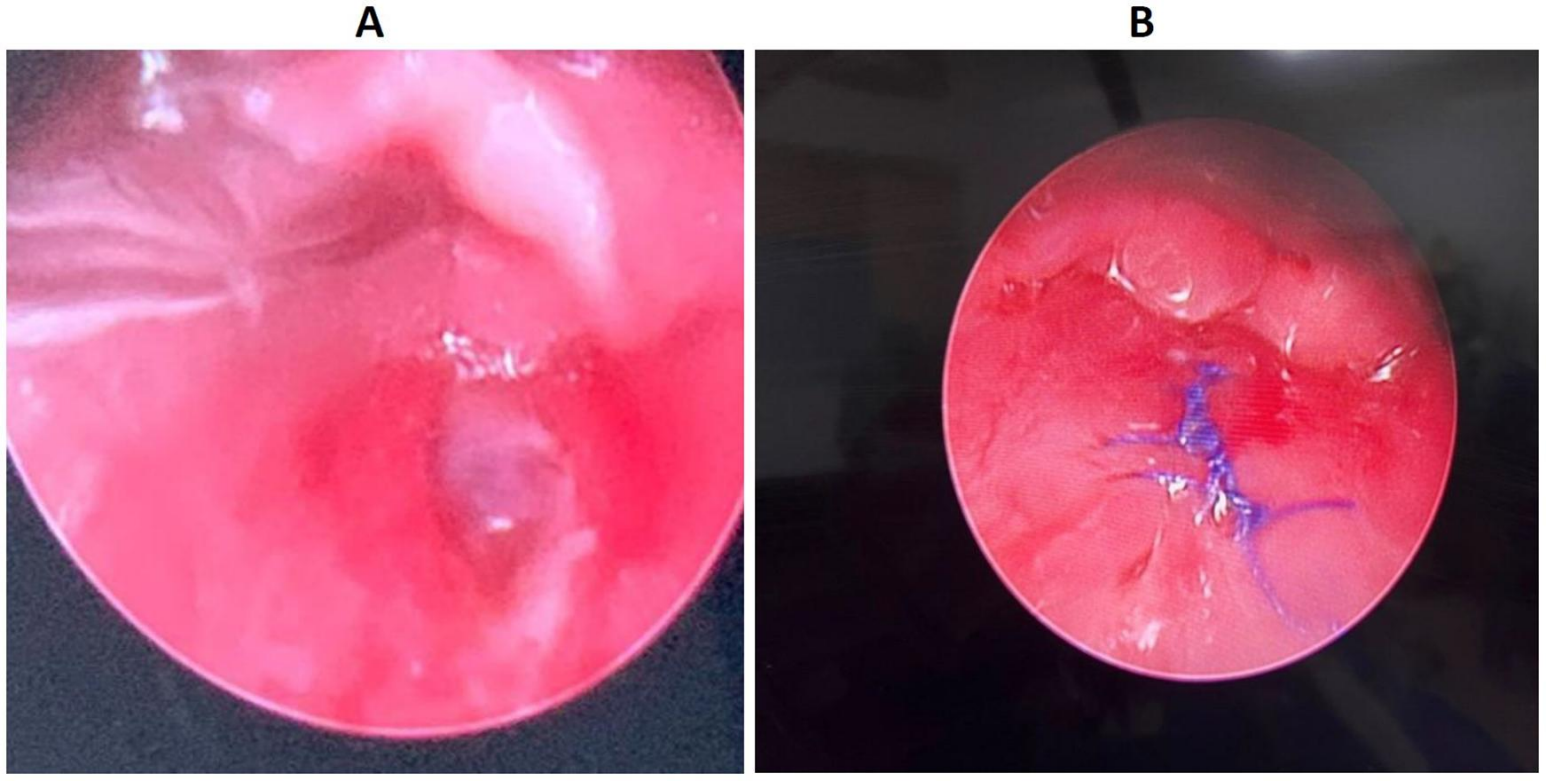
**(A)** Posterior pharyngeal wall defect. **(B)** Postsurgical closure of the defect.

Further bedside diagnostic evaluation was limited by the child’s age and level of cooperation, in addition to the suspected risk of airway compromise. Therefore, he was taken to the operating room for examination under general anesthesia to directly visualize the pharynx and esophagus for mucosal injury and to assess vascular integrity. Intraoperatively, a direct laryngoscopy performed by the otolaryngologist identified a 1.5 cm posterior pharyngeal defect, located superior to the esophageal inlet and not involving the esophagus (Figure 3A). A transoral closure of the defect was performed using 4-0 Vicryl suture (Figure 3B). Then a nasogastric tube was inserted under direct vision to provide adequate and safe nutrition. Postoperatively, the patient was kept nil per os (NPO), receiving only enteral feeds. Swallowing function was assessed with an upper gastrointestinal (GI) contrast study on the fifth postoperative day, which showed a normal esophageal structure and swallowing act. There was no hypopharyngeal contrast leak or aspiration. Subsequently, oral feeding was gradually introduced, and the nasogastric tube was removed.

The patient was discharged home on the sixth postoperative day. Unfortunately, he did not show for the clinic follow-up appointment. However, a follow-up telephone call was reassuring as he did not have dysphagia, chronic respiratory symptoms, or pneumonia. The medical record was reviewed 6 months after the incident confirmed no physician visits for feeding issues or lower respiratory tract infection.

From the patient’s family perspective, the parents were distressed about the unexpected course of the perceived minor trauma and the need to go to the operating room. They expressed difficulty accepting the seriousness of the injury until the diagnostic findings were explained. Postoperatively, after sharing the intraoperative findings and the photo, they expressed their gratitude. The parents appreciated the clear communication from the team. This incident made them more vigilant.

## Discussion

This brief report stresses the importance of cautious evaluation of oropharyngeal injury in children. Impalement injury of the oral cavity represents a distinct subset of pediatric trauma, disproportionately affecting children less than 5 years old with male predominance. Toddlers and preschool-age children tend to ambulate with objects in their mouths, risking injury once direct force is applied during a fall or a collision with another person or object. The most common impaled objects were toothbrushes, toys, sticks, pens/pencils, straws, and chopsticks in Asian countries. Injuries most frequently occurred in the soft palate (44.4%) followed by the hard palate (18.1%). The pharynx is rarely affected (3.5%), as it is anatomically protected anteriorly by the mandible (1).

Oropharyngeal impalement injuries are often benign but can lead to rare, serious complications. Initial assessment of patients should include careful evaluation of possible life-threatening complications such as airway obstruction, severe bleeding, surgical emphysema, mediastinitis, mediastinal abscess, or other deep neck space infections (3). Due to the anatomical proximity of the carotid sheath in relation to the oropharynx, impalement injury can seldom lead to dissection or thrombus of the internal carotid artery and then subsequent stroke (2).

Management should always start with airway assessment, and if compromised, secure it first with intubation. Then, hemodynamic stability should be checked and corrected adequately, especially in cases of severe bleeding. Admission is indicated for patients with imaging findings of pneumomediastinum or retropharyngeal air and suspicion of neurologic or vascular compromise (4).

A high index of suspicion should be maintained for esophageal involvement as a result of the pharyngeal trauma. The finding of neck crepitus on physical examination should trigger more diagnostic imaging to look for air leaks, subcutaneous emphysema, and pneumomediastinum. Direct laryngoscopy allows better visualization of the upper aerodigestive injury and can identify abnormal findings in 80% of patients with neck crepitus (5).

Soft tissue neck and chest imaging should be performed earlier, looking for air leaks such as subcutaneous emphysema, pneumomediastinum, or retained objects at the site of the injury. In addition, a contrast study of the esophagus, looking for local extravasation, should be performed with a water-soluble contrast to avoid local tissue and mediastinal irritation (6, 7). Computerized tomography, CT, is performed to evaluate the extent of the injury and its relation to the adjacent vascular structure. The percentage of ordering CT in impalement injuries varied in the reported case series from 11% to 49% (1, 8). Recent meta-analysis and systematic reviews for the utility of CT angiography in children with oropharyngeal trauma concluded that internal carotid injury leading to cerebrovascular accident is very low, 0.31%.

The likelihood of cerebrovascular accidents was elevated in children with injuries over 2 cm in length at the posterior pillars and tonsils, but it was diminished in injuries located in the soft palate. The risk escalates if the cause involves writing tools or sticks (9). The yield of CT angiography in oropharyngeal trauma was very low, did not improve the clinical outcome, and rarely changed the clinical management (10).

Once a perforation in the pharyngeal mucosa is confirmed, a nasogastric tube should be inserted during endoscopy, and antibiotics to be started immediately. Further surgical or conservative management of pharyngeal injury is a dilemma due to the rare nature of the injury and the lack of consensus or international guidelines. The management should be based on a multidisciplinary team decision to provide a tailored approach to the case.

A non-operative approach is increasingly advocated for impalement injuries of the oral cavity. In a large case series, nearly two-thirds of the patients were successfully managed conservatively, as most injuries tend to heal spontaneously (1, 8). Surgical intervention is generally reserved for selected cases, such as those involving the removal of retained foreign bodies, large perforations, esophageal involvement, wounds size exceeding 2 cm, hanging mucosal flap, or penetrating injuries (11, 12). However, it is important to note that the 2 cm wound-size threshold is derived from adult studies, which may not be applicable to the pediatric population, given the difference in anatomic dimensions. In fact, a large pediatric case series reported that 75% of children with pharyngeal wounds greater than 1 cm underwent surgical exploration (8). Some literature suggests that sutures may even precipitate further damage and prolong the healing process if done unnecessarily.

Empiric antibiotics are used to reduce the risk of infection and oral contamination. The rate of secondary infection is very low, ranging from 0% to 0.9%. (1, 13) In patients with pharyngeal perforation, performing a contrast study on postoperative day 5 is advocated to assess esophageal integrity. This approach was inferred from the management of esophageal perforations (6, 14).

**Figure 4:**
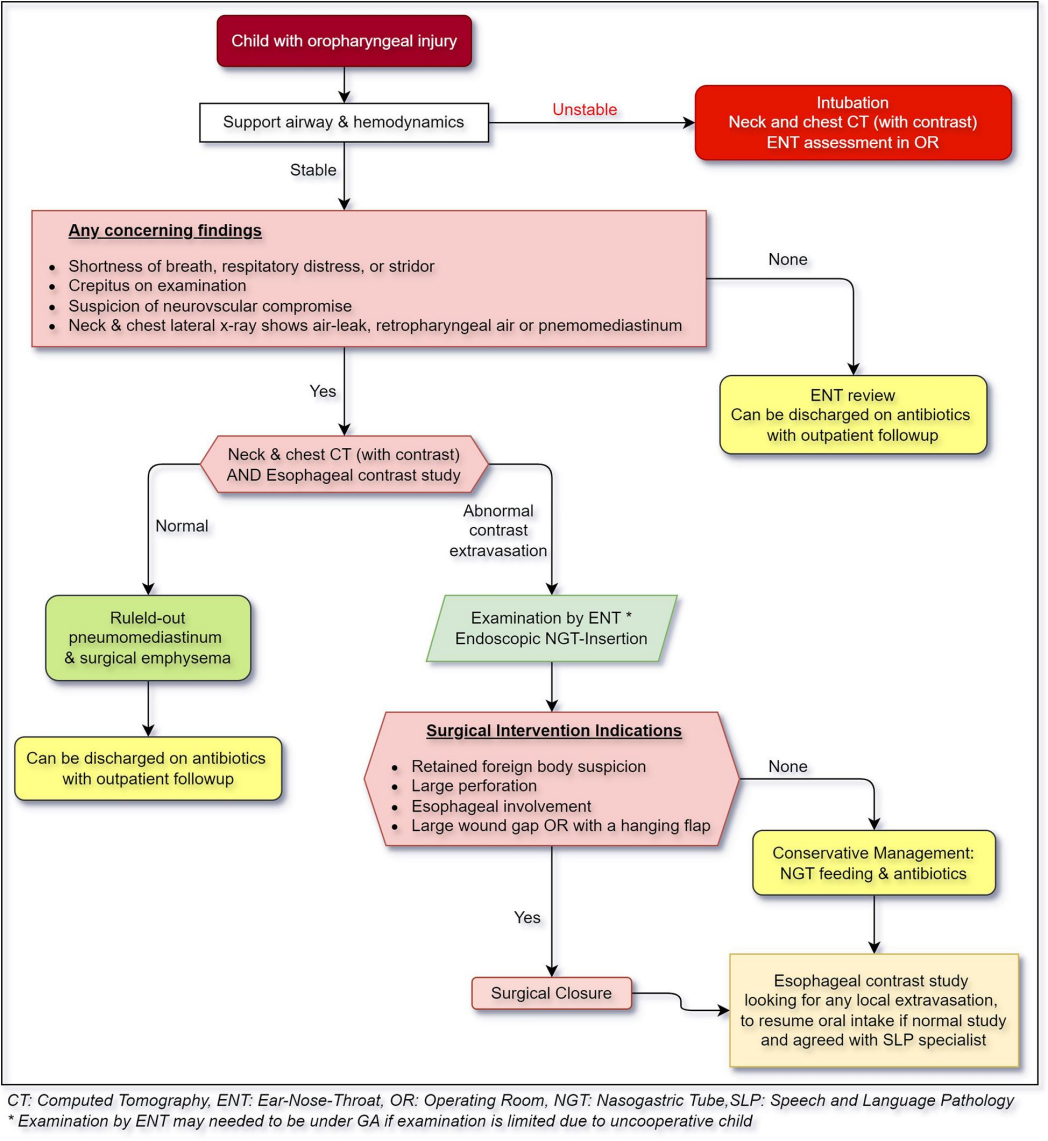
Algorithm for managing pharyngeal injury in children.

Based on the current evidence, we propose a practice algorithm for evaluation and management of pharyngeal injuries in children Figure 4.

This case report highlighted a few limitations and challenges in daily clinical assessment. For example, performing a comprehensive clinical evaluation in an agitated and uncooperative child with suspected injury is challenging. To overcome this challenge, procedural sedation became the standard of care to achieve the diagnostic and therapeutic intended outcome. The degree of sedation is dependent on several factors, such as the duration and invasiveness of the procedure, as well as the developmental status of the children and the degree of cooperation needed (15). Another challenge was not attending the follow-up appointment. However, telemedicine did overcome this challenge and provided the needed access to healthcare, which satisfied both parents and healthcare providers (16).

As in our case, clinical history and physical exam suggested an expanding trapped air within underlying soft tissues that was confirmed later by a CT scan showing air trapping all the way to the lower chest area. This mandated the admission of the patient to the intensive care unit for close airway monitoring. In our case, the patient was taken for surgical suturing due to the presence of extensive surgical emphysema impeding the patient's airway.

## Conclusion

Oropharyngeal impalement injuries are common, and the majority are self-healing. Potential complications can rarely occur, such as vascular injury and air leak. More vigilance is needed to observe serious complications, especially in an uncooperative child. The presence of crepitus and a focal injury point on physical examination should prompt more imaging and careful examination under anesthesia.

## Data Availability

The original contributions presented in the study are included in the article/Supplementary Material; further inquiries can be directed to the corresponding author.
