# Supplementary material for: Pharyngeal penetration complicated by pneumomediastinum and surgical emphysema: a rare pediatric case report
Source: Front Pediatr. 2025 Nov 18;13:1638899. doi: 10.3389/fped.2025.1638899 (PMC12669172; doi:10.3389/fped.2025.1638899)
Supplement: Supplementary file 1 [file Datasheet1.pdf]

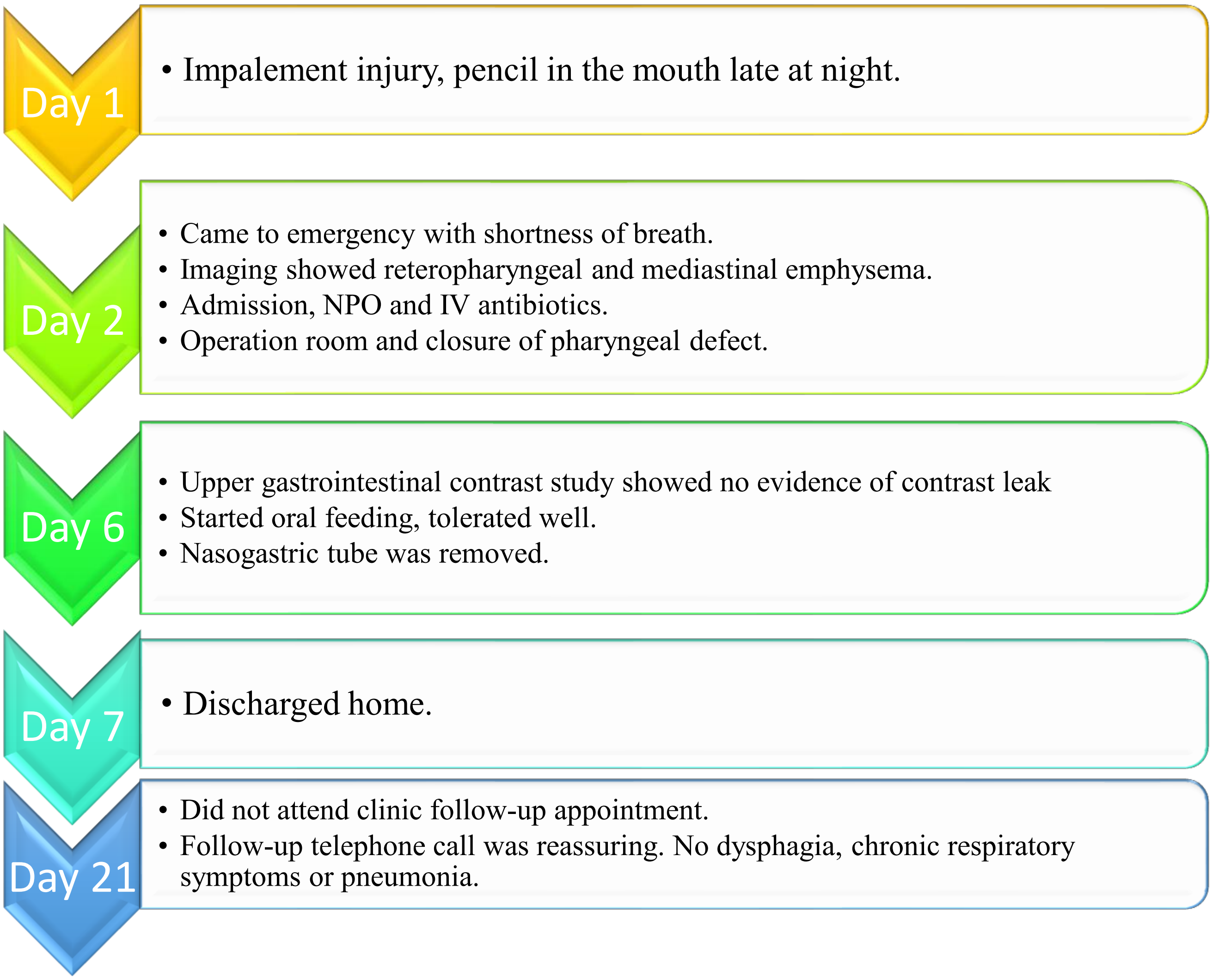

Flowchart of clinical management for pharyngeal impalement injury with mediastinal emphysema. NPO = nil per os; NG = nasogastric; GI = gastrointestinal.
